# Fail-Closed Validation of Lineage-Associated Transcript-Count Diversity After IFN-β Stimulation in a Public Human PBMC Dataset

**DOI:** 10.3390/ijms27188182

**Published:** 2026-09-14

**Authors:** Roberto Navarro Quiroz, Katherine Escorcia Lindo, Andrea Jaruffe Pinilla, Yirys Díaz-Olmos, Cecilia Fernández-Ponce, Eloina Zarate Peñata, Yesit Bello Lemus, Lisandro Pacheco Lugo, Leonardo Pacheco Londoño, Antonio Acosta Hoyos, Nataly Galan Freyle, Katy Elena Retamoza Chamorro, Jose Luis Villarreal-Camacho, Elkin Navarro Quiroz

**Affiliations:** 1 Center for Research in Critical Dynamics, Barranquilla 080002, Colombia; robertcnavarro@gmail.com; 2 Facultad de Ingeniería, Tecnológico de Antioquia—Institución Universitaria, Medellín 050036, Colombia; 3 Centro de Investigaciones en Ciencias de la Vida (CICV), Facultad de Ciencias Básicas y Biomédicas, Universidad Simón Bolívar, Barranquilla 080002, Colombia; katherineescorcia52@gmail.com (K.E.L.); andrea.jaruffe@unisimon.edu.co (A.J.P.); eloina.zarate@unisimon.edu.co (E.Z.P.); yesit.bello@unisimon.edu.co (Y.B.L.); lisandro.pacheco@unisimon.edu.co (L.P.L.); leonardo.pacheco@unisimon.edu.co (L.P.L.); antonio.acosta@unisimon.edu.co (A.A.H.); nataly.galan@unisimon.edu.co (N.G.F.); 4 Programa de Medicina, División de Ciencias de la Salud, Universidad del Norte, Barranquilla 081007, Colombia; yirysdiaz@gmail.com; 5 Instituto de Investigación e Innovación Biomédica de Cádiz (INiBICA), 11009 Cádiz, Spain; ceciliamatilde.fernandez@uca.es; 6 Departamento de Biomedicina, Biotecnología y Salud Pública, Facultad de Medicina, Universidad de Cádiz, 11003 Cádiz, Spain; 7 Facultad de Ciencias de la Salud, Universidad de la Costa–CUC, Barranquilla 080002, Colombia; kretamoz2@cuc.edu.co; 8 Facultad de Ciencias de la Salud, Exactas y Naturales, Universidad Libre, Barranquilla P.O. Box 1752, Colombia; josevillarrealc@gmail.com

**Keywords:** single-cell RNA sequencing, Shannon entropy, interferon-beta, PBMC, rarefaction, pseudobulk, donor-aware inference, technical validation

## Abstract

Shannon entropy of single-cell transcript counts reflects composition and sampling. We tested whether interferon-beta (IFN-β) stimulation was associated with donor-consistent diversity changes after employing technical controls. We reanalyzed GSE96583 peripheral blood mononuclear cells from eight donors with lupus, treating donors—not the 8949 target cells obtained from them—as inferential units. Analyses included sampling without replacement at 562 unique molecular identifiers, a 750-molecule sensitivity analysis, Miller–Madow correction, IFN-gene removal, and exact compositional decomposition. Mean paired-donor raw-count plug-in entropy increased by 0.138 bits in CD14+ monocytes and 0.329 bits in FCGR3A+ monocytes and decreased by 0.080 bits in natural killer cells. Directions persisted across prespecified robustness variants. Monocyte increases accompanied higher evenness and lower transcript dominance; natural killer cells showed the opposite pattern, without consistent richness changes. Adding IFN activity to donor-, condition-, and technical-covariate-adjusted models increased R^2^ by at most 0.0108. Entropy contributions strongly overlapped pseudobulk expression changes. In GSE194122, the implemented binary chromatin-accessibility entropy was determined by open-peak count, precluding biological cross-modality coupling claims. These findings support a lineage-divergent association within this single lupus cohort; independent replication is required before broader biological or diagnostic interpretation.

## 1. Introduction

Single-cell RNA sequencing describes each cell through a finite vector of transcript counts. After division by a cell’s total count, this vector can be treated as an observed composition. Shannon entropy then summarizes how evenly the observed transcript mass is distributed among genes [1]. This is a within-cell property: it is not the number of cell states in a sample, nor does it by itself establish functional flexibility, differentiation potency, or a regulatory mechanism. Entropy-derived quantities have been used in single-cell biology for several distinct purposes [2], making precise definition of the observable and the unit of interpretation essential.

For cell *c*, the usual plug-in estimator is computed from observed unique molecular identifier (UMI) proportions. This estimator is affected by the finite number of sampled molecules and by the number of detected categories [3]. In droplet single-cell data, library size, detection, and zeros are integral features of the UMI sampling process rather than nuisances that can be ignored or uniformly interpreted [4,5,6,7,8,9]. Consequently, a comparison of plug-in entropy across conditions can change because of sampling depth, the appearance or disappearance of low-count genes, or altered dominance by a few abundant transcripts. An analysis that reports only entropy cannot distinguish these possibilities.

The nested design of single-cell experiments adds a second problem. Cells obtained from the same donor are subsamples, not independent biological replicates. Cell-level inference that ignores this dependence can greatly overstate precision [10,11]. Multi-sample methods and pseudobulk approaches address the same principle by preserving between-subject variability [11,12]. For an experimental contrast applied to multiple people, the donor should therefore determine the inferential sample size even when cell-level quantities are computed first.

GSE96583 is a public 10x Chromium peripheral blood mononuclear cell (PBMC) dataset with matched control and interferon-beta (IFN-β)-stimulated samples from eight donors with lupus [13]. It provides a useful setting in which to test whether transcript-count diversity changes within annotated lineages after stimulation. The original analysis reported increases in monocytes and a decrease in natural killer (NK) cells, but its nominal 5000-UMI “normalization” was a deterministic cap that left almost every cell unchanged. It also used a pooled-cell IFN-score model without donor and condition terms, interpreted module entropy without separating module zeros and abundance, and treated entropy contributions as more distinct from differential expression than the data supported. In addition, configured gene symbols were not completely aligned with their Ensembl identifiers. These issues required a prospective, auditable reanalysis rather than a textual clarification alone.

**Figure 1:**
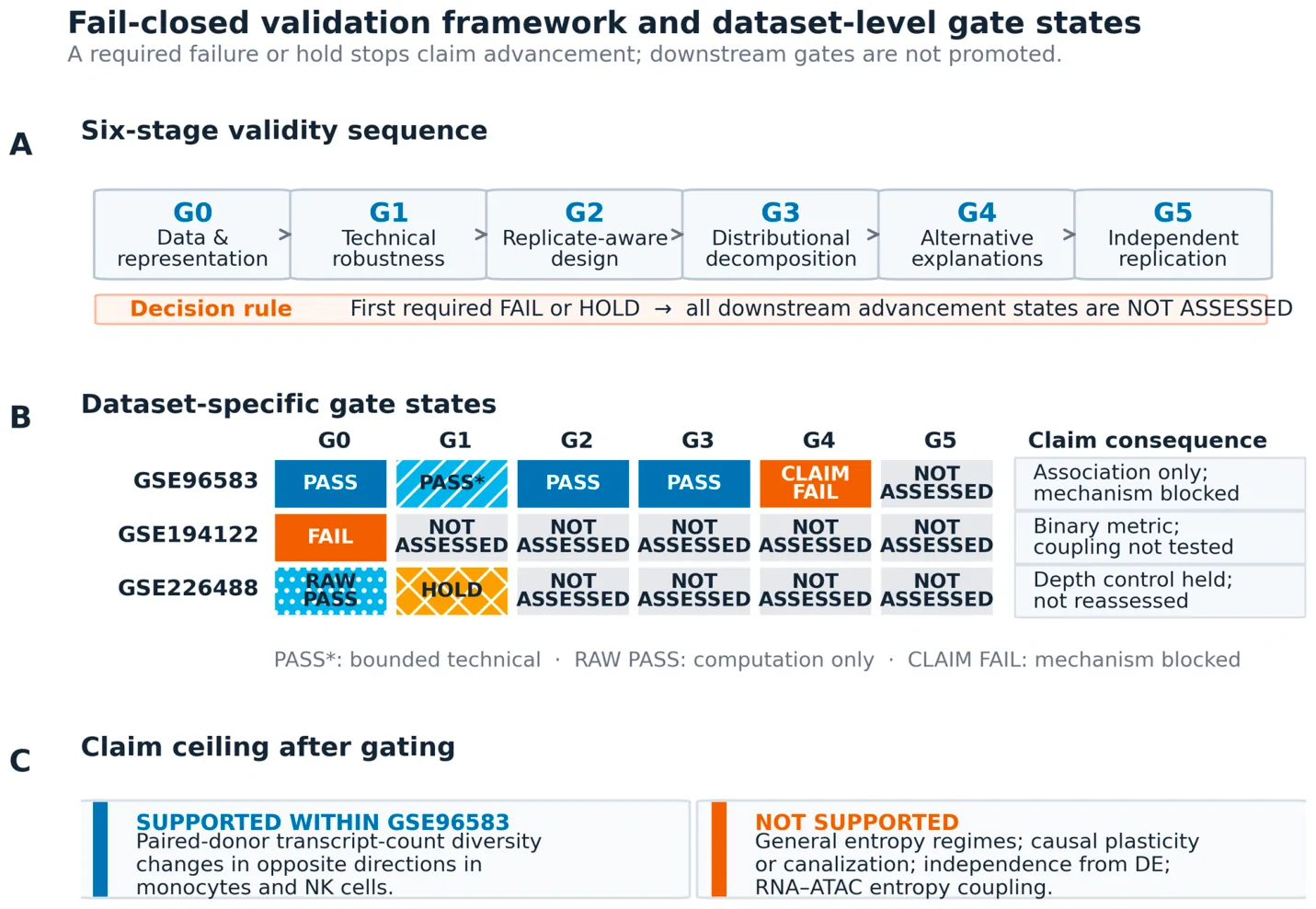
Fail-closed validation sequence and dataset-specific gate states. (**A**) Six required gates progress from representation validity to external replication. The first required failure or hold stops claim advancement. (**B**) GSE96583 passed G0–G3, but G4 blocked mechanistic interpretation and G5 was not assessed; GSE194122 failed G0 for binary ATAC entropy; GSE226488 retained a computation-only raw pass at G0 and was held at G1 because the historical depth cap was invalid. PASS*, bounded technical pass; RAW PASS, computation-only pass; CLAIM FAIL, failure of mechanistic claim advancement; HOLD, reassessment not completed; NOT ASSESSED, no downstream advancement after a required failure or hold. (**C**) Supported and unsupported claim classes. All gate states are defined in Supplementary Table S6. In (**B**), blue denotes PASS; diagonally hatched light blue, PASS*; dotted light blue, RAW PASS; vermilion, FAIL or CLAIM FAIL; cross-hatched gold, HOLD; and gray, NOT ASSESSED. In (**C**), blue and vermilion distinguish supported and unsupported claim classes, respectively.

We therefore implemented a six-stage fail-closed sequence (Figure 1): data and representation validity (G0), technical robustness (G1), replicate-aware design (G2), distributional decomposition (G3), alternative-explanation stress tests (G4), and external replication (G5). A required failure stops claim advancement; downstream diagnostics cannot retroactively convert that failure into a pass. GSE96583 was the primary dataset. GSE194122 [14] served not as an external replication but as a falsification demonstration of the fail-closed sequence: auditing the accessibility representation before testing any association between RNA and assay for transposase-accessible chromatin (ATAC) data showed that binary ATAC entropy contained no information beyond open-peak count, thereby preventing a technical count relationship from being misinterpreted as biological cross-modality coupling. The historical GSE226488 analysis was placed on hold because its depth control was invalid and the metadata required for donor- and lineage-aware reassessment were unavailable. The objective was not to establish a general biological theory from entropy, but to determine which bounded statements remain supported after correcting the analysis.

## 2. Results

### 2.1. A Prospective Decision Lock and Identifier Audit Defined the Corrected Analysis

Before inspecting the corrected revision outputs, we fixed the donor as the unit of inference, the stimulated-minus-control contrast within each donor and lineage, the target lineages (CD14+ monocytes, FCGR3A+ monocytes, and NK cells), the entropy estimators, the rarefaction depths, the IFN-removal operations, the module estimands, the matched-null design, and the claim ceiling. The maximum possible claim was a hypothesis-generating association within one public dataset; GSE194122 and NK cells were not designated as external replications (Figure 1A–C).

An outcome-blind audit compared every configured symbol–Ensembl pair with the recovered GSE96583 gene map. Of 141 configuration rows, 119 were exact, 18 incorrect row assignments were repaired by a unique exact symbol match, and four CXCL8/IL8 occurrences were retained as accepted historical aliases; no mapping remained unresolved (Supplementary Figure S2C; Supplementary Table S6). Outputs generated before this repair were quarantined and excluded from every number reported below. The corrected configuration contained all genes required for the primary and sensitivity sets.

### 2.2. The Historical 5000-UMI Cap Was Not an Equal-Depth Control

**Figure 2:**
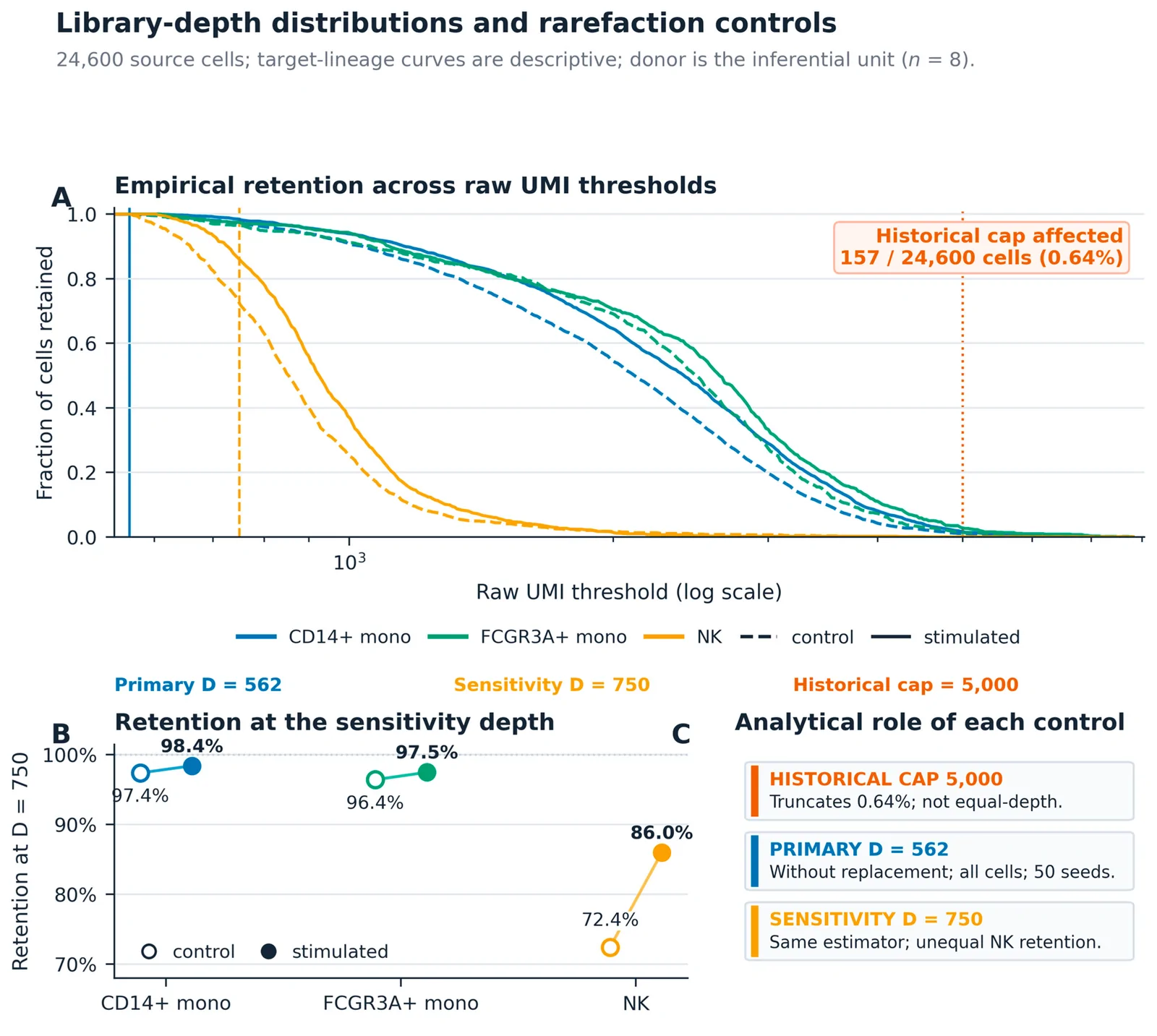
Audit and correction of depth control. (**A**) Empirical fractions of target-lineage cells retained across raw UMI thresholds; lineage is encoded by color, and condition by line style. Vertical lines identify the primary rarefaction depth (562 UMIs), sensitivity depth (750 UMIs), and historical 5000-UMI cap. The historical operation changed only 157/24,600 source cells. (**B**) Condition-specific retention at 750 UMIs, shown as connected control and stimulated values within each lineage; the NK imbalance motivated sensitivity-only interpretation. (**C**) Operational distinction among the invalid cap, primary sampling without replacement, and sensitivity analyses. ctrl, control; stim, IFN-β-stimulated. Blue, green and gold identify CD14+ monocytes, FCGR3A+ monocytes and NK cells. Dashed and solid curves indicate control and stimulated cells; open and filled circles in B indicate the corresponding conditions.

The recovered GSE96583 object contained 24,600 cells and 35,635 gene features. The corrected target analysis included 5375 CD14+ monocytes (2843 control; 2532 stimulated), 1591 FCGR3A+ monocytes (756 control; 835 stimulated), and 1983 NK cells (935 control; 1048 stimulated), amounting to 8949 cells from eight paired donors. Only 157 of the 24,600 source cells exceeded 5000 UMIs; the historical cap therefore changed only those cells and did not equalize depth for the remainder of the analysis (Figure 2A). It was removed as evidence for technical robustness.

The primary correction sampled 562 molecules without replacement from each cell, the minimum whole-transcriptome depth in the source object, and therefore retained all 24,600 cells. For each cell, sampling was repeated 50 times using reproducible seeds, and the resulting estimates were averaged; the formal sampling distribution and seed construction are specified in the Methods section. A 750-UMI sensitivity analysis retained 97.4% and 98.4% of control and stimulated CD14+ monocytes, 96.4% and 97.5% of FCGR3A+ monocytes, and 72.4% and 86.0% of NK cells, respectively (Figure 2B; Supplementary Figure S1A). Because the NK retention was condition-imbalanced, 750 UMIs was not promoted to the primary depth.

We also examined the corrected 38-gene IFN union in both operational orders. In the first, the complete transcriptome was rarefied to 562 UMIs, IFN-union counts were removed, and the residual composition was renormalized. In the second, IFN-union counts were removed from raw counts before rarefaction to the prospectively fixed 503-UMI sensitivity depth. After the identifier repair, this second operation retained 8946/8949 target cells (99.97%); three cells below 503 residual UMIs were excluded rather than imputed (Supplementary Table S1).

### 2.3. Donor-Paired Entropy Directions Persisted Across Depth and Finite-Count Controls

On raw counts, the mean paired-donor change in plug-in entropy was +0.138 bits for CD14+ monocytes (95% donor-bootstrap interval, +0.075 to +0.201; seven out of eight donors positive), +0.329 bits for FCGR3A+ monocytes (+0.287 to +0.372; eight out of eight positive), and −0.080 bits for NK cells (−0.103 to −0.057; eight out of eight negative). The corresponding medians were +0.137, +0.337, and −0.070 bits. These are donor summaries; the thousands of cell-level observations were not treated as independent replicates.

**Figure 3:**
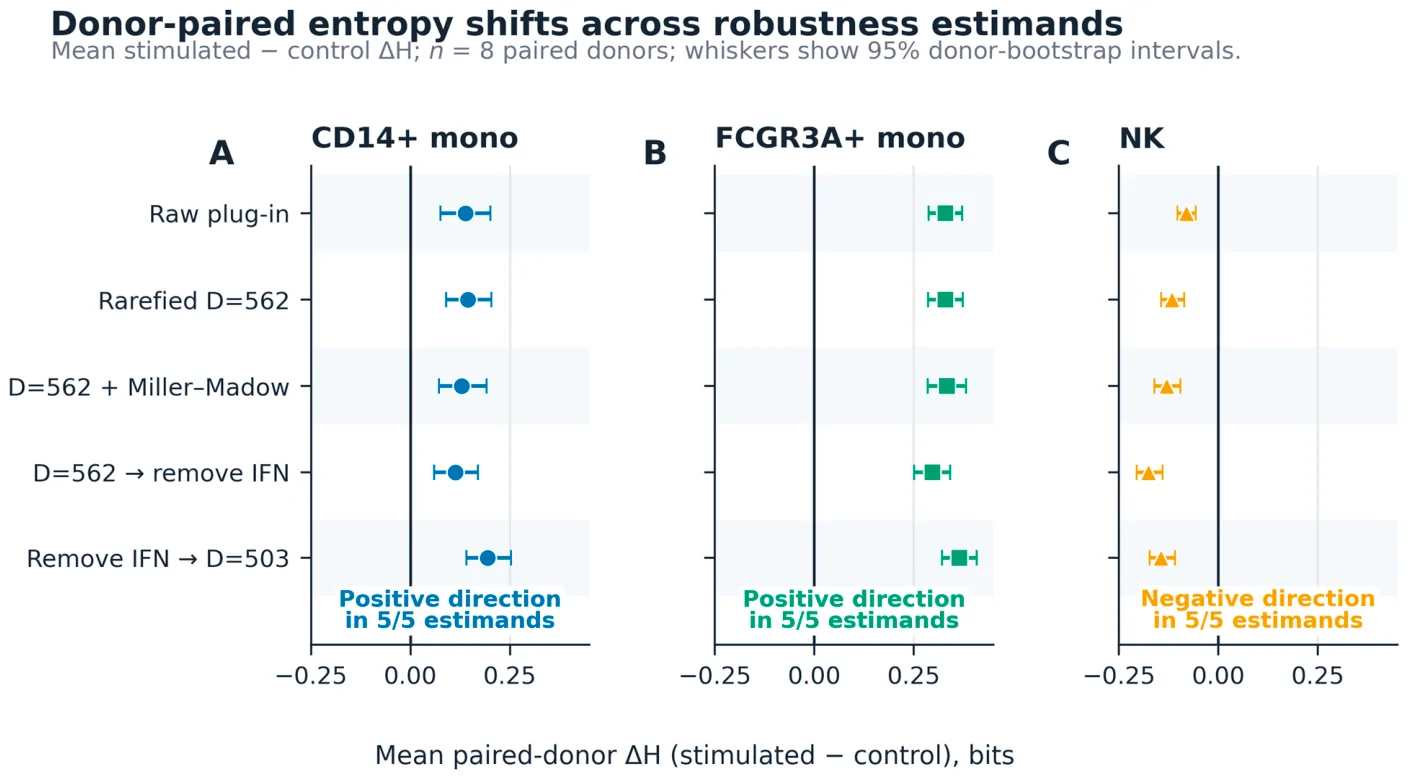
Donor-paired transcript-count entropy differences across robustness variants. Mean stimulated-minus-control ΔH and 95% donor-bootstrap intervals for CD14+ monocytes (**A**), FCGR3A+ monocytes (**B**), and NK cells (**C**). All panels use the same horizontal scale. Variants are raw plug-in entropy, 562-UMI hypergeometric rarefaction, rarefaction with Miller–Madow correction, rarefaction followed by corrected IFN-union removal, and IFN-union removal followed by 503-UMI rarefaction. The unit of inference is the donor (*n* = 8). MM, Miller–Madow. The arrow (→) means “followed by” and indicates processing order, not a causal relationship.

The direction was unchanged in raw Miller–Madow estimates and in the four primary corrected variants: 562-UMI rarefaction, rarefaction plus Miller–Madow correction, rarefaction followed by IFN removal, and IFN removal followed by 503-UMI rarefaction (Table 1; Figure 3; Supplementary Figure S1B–D). Across the five primary variants, including raw plug-in entropy, at least seven out of eight donors had the mean direction in each monocyte lineage; all eight were concordant in the four corrected variants. NK cells retained the opposite direction in all eight donors across all five variants. The results at 750 UMIs also had the same signs (+0.138, +0.322, and −0.136 bits for CD14+, FCGR3A+, and NK cells), but were interpreted as sensitivity results because of unequal retention.

### 2.4. Evenness and Dominance, Rather than Richness, Characterized the Primary Differences

**Figure 4:**
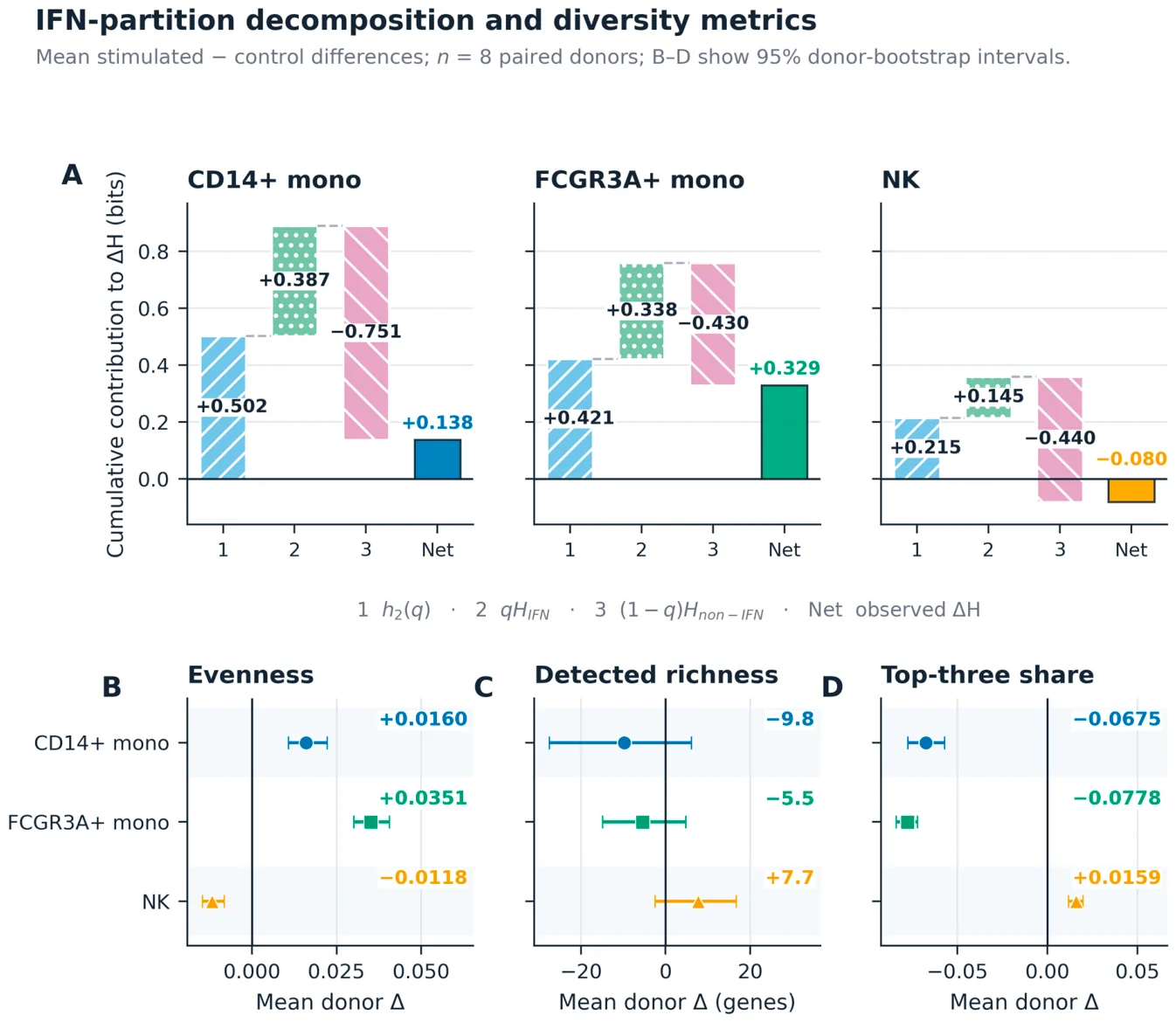
Exact IFN/non-IFN decomposition and companion diversity metrics. (**A**) Lineage-specific waterfalls for the mean paired-donor contributions of the binary IFN-partition term h_2_(q), weighted within-IFN entropy qH(IFN), and weighted non-IFN entropy (1 − q)H(non-IFN), where q is the IFN transcript fraction; the three numbered steps sum to the directly observed net ΔH bar. (**B**) Normalized evenness change. (**C**) Detected-gene richness change. (**D**) Top-three transcript-share change. Error bars in B–D are 95% donor-bootstrap intervals. Every Δ is stimulated minus control. In (**B**–**D**), blue circles, green squares and gold triangles identify CD14+ monocytes, FCGR3A+ monocytes and NK cells, respectively. In (**A**), blue diagonally hatched, green dotted and pink diagonally hatched bars represent the binary-partition, weighted within-IFN and weighted non-IFN terms; solid lineage-colored bars show the observed net change.

The entropy changes were not accompanied by a consistent donor-level change in detected-gene richness. Mean stimulated-minus-control richness was −9.8 genes in CD14+ monocytes (95% interval, −27.5 to +6.1), −5.5 in FCGR3A+ monocytes (−15.0 to +4.8), and +7.7 in NK cells (−2.5 to +16.7); every interval included zero (Figure 4C; Supplementary Table S2).

In contrast, normalized evenness increased in both monocyte lineages (+0.0160 and +0.0351; eight out of eight donors) and decreased in NK cells (−0.0118; eight out of eight; Figure 4B). The effective number of expressed genes, 2^H^, increased by 13.0 in CD14+ and 48.8 in FCGR3A+ monocytes and decreased by 13.6 in NK cells. Monocyte top-one shares decreased by 0.059 and 0.069, and top-three shares decreased by 0.067 and 0.078. In NK cells, the top-one change was small and inconsistent (+0.0022; interval crossing zero), whereas the top-three share increased by 0.0159 in eight out of eight donors (Figure 4D). Thus, the primary observation concerns redistribution of observed transcript shares and dominance, not a consistent gain or loss of detected genes.

### 2.5. Exact Decomposition Separated IFN-Associated and Non-IFN Contributions

For the corrected 38-gene IFN union, the entropy chain rule reconstructed whole-transcriptome entropy to numerical precision (maximum absolute cell-level error, 7.8 × 10^−15^ bits). Stimulation increased the IFN count fraction by 0.1229 in CD14+ monocytes, 0.1005 in FCGR3A+ monocytes, and 0.0399 in NK cells. The between-partition term and the weighted within-IFN term were positive in all three lineages. The weighted non-IFN term was negative in all three (Table 2; Figure 4A).

In CD14+ monocytes, the partition (+0.502 bits) and weighted IFN (+0.387) terms exceeded the negative weighted non-IFN term (−0.751), reconstructing +0.138 bits. In FCGR3A+ monocytes, +0.421 and +0.338 exceeded −0.430, reconstructing +0.329 bits. In NK cells, +0.215 and +0.145 did not exceed −0.440, reconstructing −0.080 bits. The NK result is therefore not explained by absence of an IFN-associated component: the IFN terms were positive, but a larger negative non-IFN component determined the net direction.

### 2.6. IFN Composition Differed by Lineage, but Adjusted IFN Activity Explained Little Additional Entropy Variance

The 15-gene primary IFN set was dominated by a limited subset of transcripts after stimulation, with different compositions across lineages (Supplementary Figure S2A; Supplementary Table S3). ISG15 accounted for a mean 47.2% of IFN-set UMIs in CD14+ monocytes, 42.7% in FCGR3A+ monocytes, and 38.1% in NK cells. CXCL10 accounted for 27.2% and 27.6% in the two monocyte lineages but only 1.39% in NK cells. NK IFN-set counts were distributed more prominently among IFI6 (14.4%), IFIT3 (11.0%), IFIT1 (7.52%), MX1 (6.73%), IRF7 (4.69%), OAS1 (4.10%), and IFIT2 (3.91%). A transparent activity measure based on fixed IFN-set counts increased in eight out of eight donors in every lineage after removal of ISG15, IFIT1, or MX1 individually, and after their joint removal (Supplementary Figure S2B). No single reviewer-nominated dominant gene was necessary for the direction of this activity contrast.

However, IFN activity did not support a claim of entropy information partly independent of the stimulation response. Models containing donor fixed effects, condition, and endpoint-appropriate technical covariates were fitted before adding the fixed IFN-activity term. The maximum added R^2^ was 0.0108 (FCGR3A+ raw entropy; partial *r* = 0.185). Added R^2^ values were 0.00787 for CD14+ raw entropy and 0.00126 after 562-UMI rarefaction; the corresponding FCGR3A+ values were 0.01079 and 0.00131. NK values were near zero. Correlations between donor-level entropy and IFN-activity contrasts varied by lineage and endpoint and were interpreted descriptively because only eight paired donors were available (Supplementary Table S3). The previous pooled-cell independence gate, which omitted donor and condition, was therefore retired rather than reinterpreted.

### 2.7. Entropy-Contribution Shifts Overlapped Strongly with Paired Pseudobulk Expression Changes

**Figure 5:**
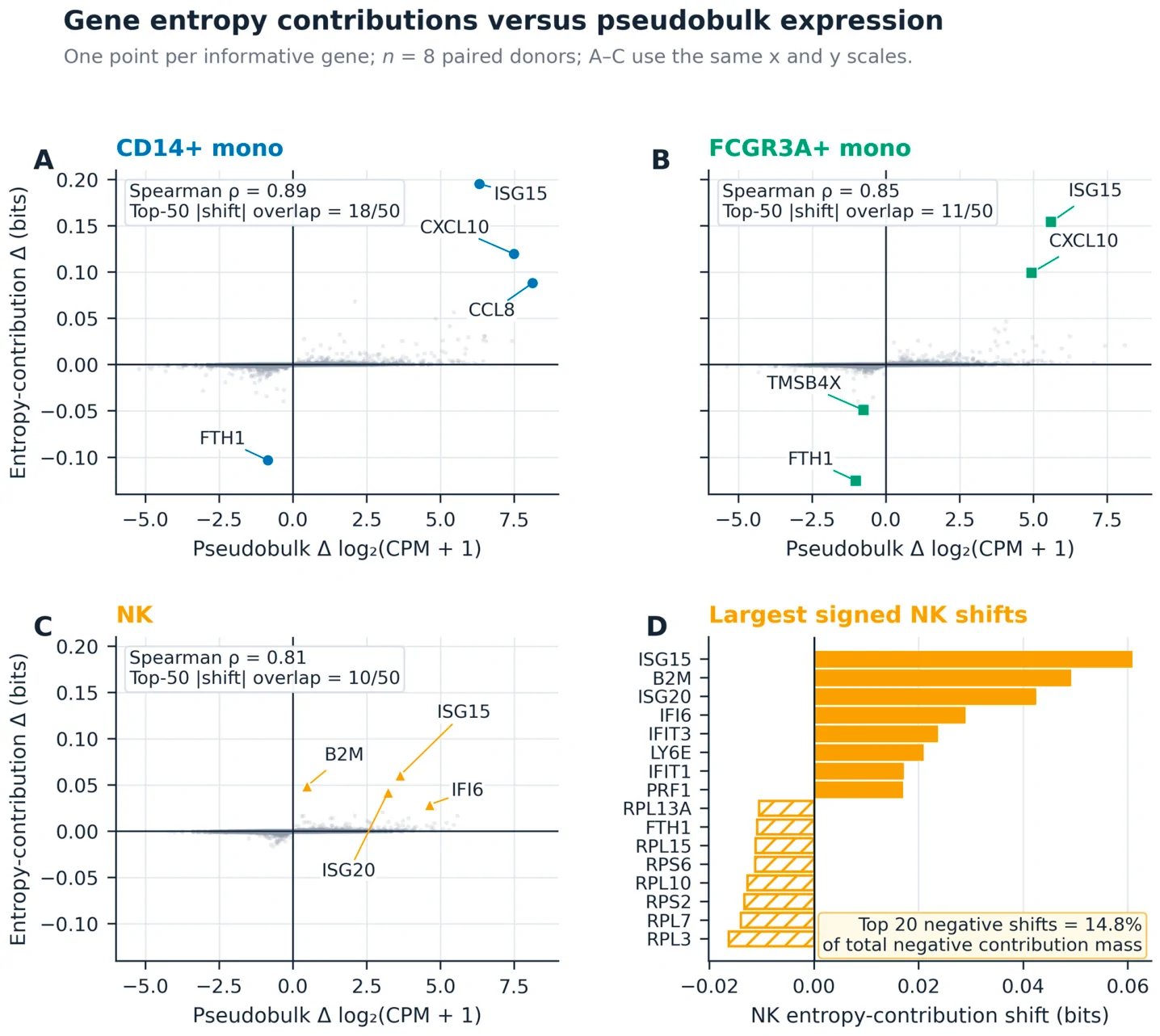
Relationship between gene-level entropy-contribution shifts and paired pseudobulk expression shifts. Shared-scale scatterplots for CD14+ monocytes (**A**), FCGR3A+ monocytes (**B**), and NK cells (**C**) compare mean paired-donor gene contribution changes with mean paired-donor log_2_(CPM + 1) changes; gray points are informative genes and colored, labeled points are the four largest absolute entropy-contribution shifts in each lineage. Spearman ρ and overlap of the 50 largest absolute shifts are displayed. (**D**) Largest positive and negative NK entropy-contribution shifts; fill and position distinguish sign. The 20 largest negative shifts account for 14.8% of total negative contribution mass.

For each gene, we decomposed a cell’s entropy into −p log_2_p contributions and compared mean paired-donor contribution shifts with mean paired-donor log_2_(CPM + 1) shifts from donor-by-lineage-by-condition pseudobulks, where CPM denotes counts per million. Rank correlations were high: ρ = 0.894 for CD14+ monocytes, 0.852 for FCGR3A+ monocytes, and 0.806 for NK cells (Figure 5A–C). The 50 largest absolute shifts overlapped by 18, 11, and 10 genes, respectively. Entropy contributions and expression change are different summaries, but both are transformations of the same counts; these results do not establish a separate biological layer independent of conventional expression analysis.

The complete NK contribution distribution answered whether its negative total shift was attributable to a few selected genes (Figure 5D; Supplementary Table S4). The 20 largest negative shifts accounted for only 14.8% of total negative contribution mass, indicating a diffuse pattern. The largest negative entries were mainly ribosomal genes (including RPL3, RPL7, RPS2, RPL10, RPS6, RPL15, RPL13A, RPL13, RPS3, RPS3A, RPS4X, RPS19, RPS7, and RPL6), together with FTH1, PTMA, CYBA, FGFBP2, TMSB4X, and NKG7. Positive NK contributions included ISG15, B2M, ISG20, IFI6, IFIT3, LY6E, IFIT1, PRF1, HLA-B, GNLY, and MX1. The presence of PRF1 and GNLY among positive contributions and NKG7 among negative contributions also argues against reducing the total entropy direction to a small, coherent cytotoxic program. We therefore report an opposite net diversity response in NK cells within GSE96583, without assigning a causal or functional label.

### 2.8. Module Conclusions Depended on Detection, Abundance, and Aggregation Level

**Figure 6:**
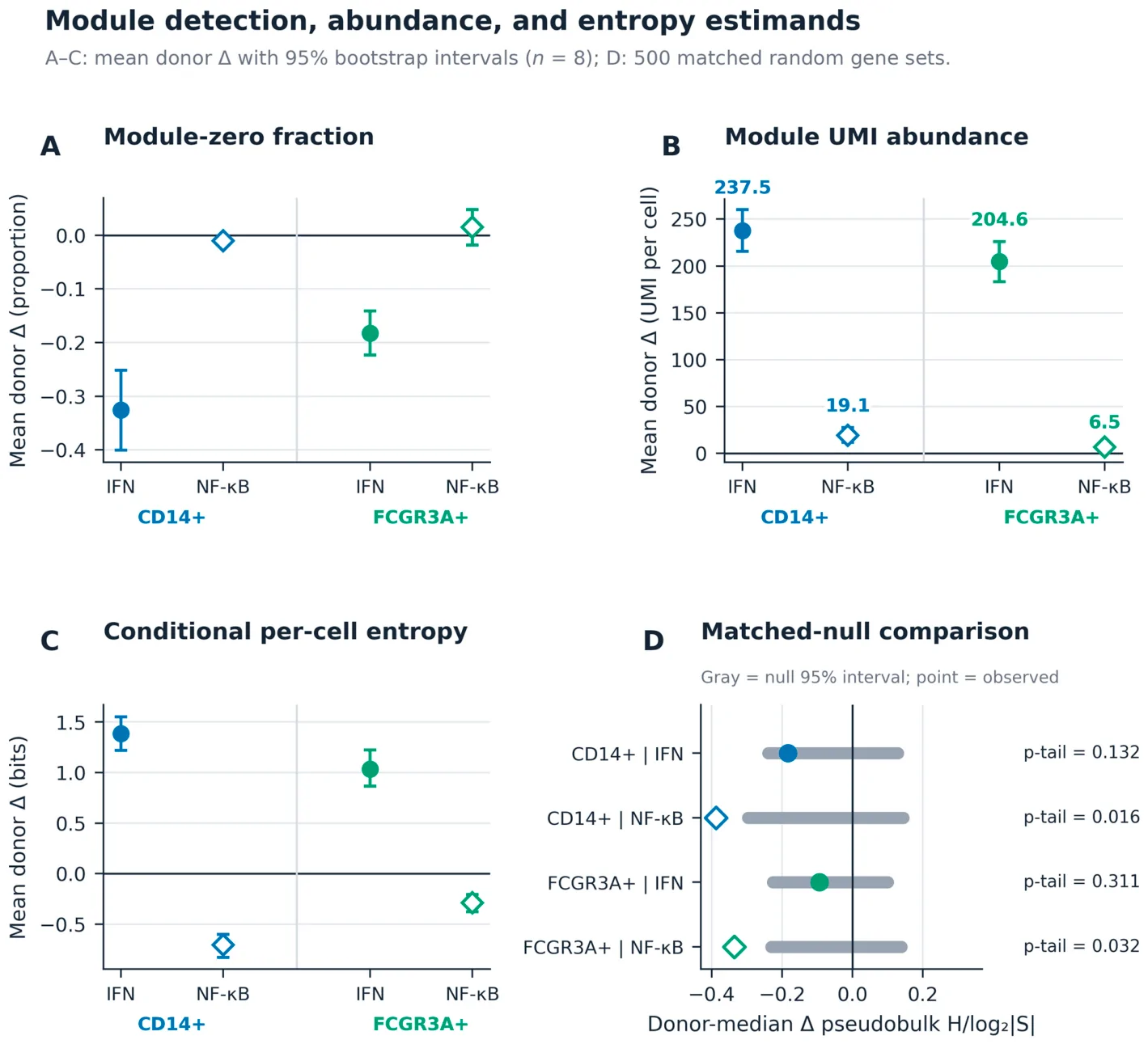
Two-part module sensitivity and matched-null analysis. Paired-donor changes in module-zero fraction (**A**), module UMIs per cell (**B**), and per-cell entropy conditional on positive module counts (**C**) for the primary IFN and inflammatory/NF-κB modules in two monocyte lineages. (**D**) Observed median donor-paired change in pseudobulk entropy normalized by log_2_ module size versus 95% intervals from 500 expression-, detection-, and size-matched random gene sets; empirical two-sided tail areas are shown. Module detection, abundance, conditional cell entropy, and pseudobulk entropy are separate estimands. Filled circles denote the primary IFN module and open diamonds the inflammatory/NF-κB module; blue and green identify CD14+ and FCGR3A+ monocytes. In (**A**–**C**), whiskers are 95% donor-bootstrap intervals. In (**D**), points show observed median paired-donor changes and gray horizontal segments show 95% matched-null intervals.

Module analysis was reformulated as a two-part problem. We first quantified the fraction of module-zero cells and module UMI abundance, and only then estimated entropy conditional on a positive module count. We separately computed donor-pseudobulk within-module entropy normalized by log_2_ module size (Figure 6; Supplementary Table S5).

For the 15-gene primary IFN module, stimulation reduced the zero-cell fraction by 0.326 in CD14+ and 0.182 in FCGR3A+ monocytes and increased mean module UMIs per cell by 237.5 and 204.6. Conditional per-cell module entropy increased by 1.383 and 1.034 bits, and entropy normalized by nominal module size increased by 0.354 and 0.265. Yet donor-pseudobulk normalized entropy decreased by 0.180 and 0.084 (median changes, −0.183 and −0.094). The per-cell and pseudobulk results answer different questions: the former describes the distribution of module counts among genes within individual cells conditional on positive module counts, in the context of a large abundance change, whereas the latter describes the distribution of aggregated module counts within each donor and condition.

For the nine-gene inflammatory/NF-κB module, mean UMIs per cell increased by 19.1 in CD14+ and 6.5 in FCGR3A+ monocytes, whereas conditional per-cell entropy decreased by 0.704 and 0.287 bits. Donor-pseudobulk normalized entropy decreased by 0.398 and 0.327. In 500 expression-, detection-, and size-matched random sets per lineage, the two-sided empirical tail areas for the IFN module were 0.132 and 0.311, so the IFN pseudobulk statistic was not exceptional in both monocyte lineages. The inflammatory/NF-κB tail areas were smaller (0.016 and 0.032), although these descriptive null comparisons cannot establish the pathway mechanism or raise the single-dataset claim ceiling (Figure 6D). Expanded IFN, TNF/NF-κB, and inflammatory gene sets preserved the broader discrepancy between conditional per-cell and donor-pseudobulk estimands (Supplementary Figure S3). Accordingly, we do not characterize the module results as a cross-validated process distinct from abundance or differential expression.

### 2.9. Binary ATAC Entropy Failed the Representation Gate, and No Dataset Supported Generalization

**Figure 7:**
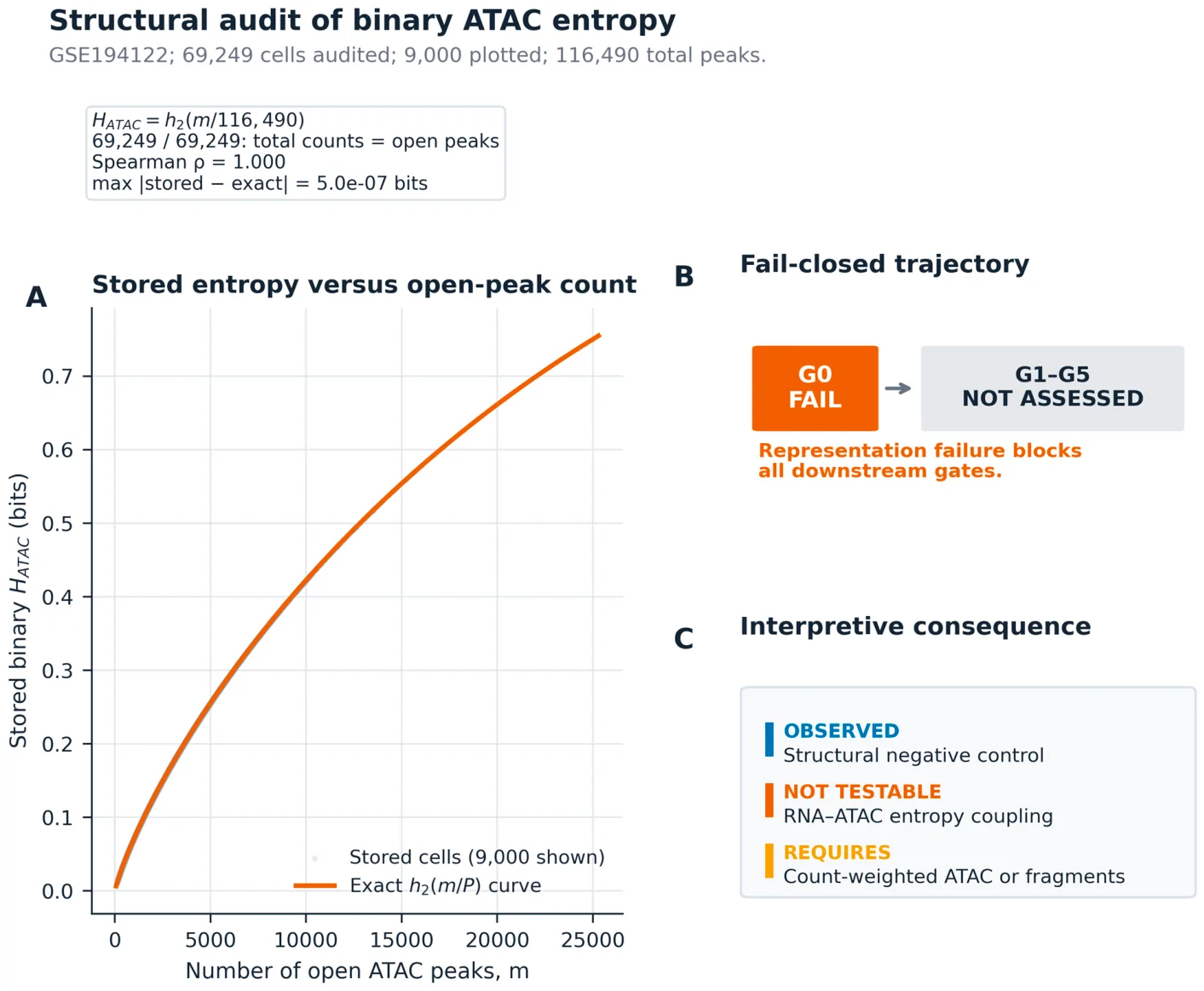
Structural limitation of binary ATAC entropy in GSE194122. (**A**) Stored ATAC entropy versus open-peak count for a seeded sample of 9000 of 69,249 audited cells, with the exact binary-entropy curve. The total ATAC count equaled the open-peak count in 69,249/69,249 cells, and Spearman ρ was 1. (**B**) G0 representation failure forced G1–G5 to adopt not-assessed states for cross-modal claim advancement. (**C**) The result is a structural negative control; count-weighted fragments or another independently varying accessibility observable would be required to test cross-modal entropy coupling.

The GSE194122 ATAC audit tested the exact observable before any cross-modal association analysis. In all 69,249 cells, the stored ATAC total count equaled the number of open peaks. A sample of matrix rows contained only the nonzero value 1, confirming the binary representation. With 116,490 total peaks and m_c_ open peaks in cell c, the computed ATAC entropy was the binary entropy h_2_(m_c_/116,490); its Spearman correlation with open-peak count was 1.000, and the maximum difference from the stored rounded values was <5 × 10^−7^ bits (Figure 7A; Supplementary Table S6). Because the metric was a deterministic transformation of open-peak count, G0 failed for the proposed cross-modal entropy question. G1–G5 were marked as not assessed for claim advancement. This is a structural negative control, not evidence for the presence or absence of biological RNA–ATAC coordination (Figure 7B,C).

The final gate states are summarized in Figure 1B and Supplementary Table S6. GSE96583 passed tests of data validity, bounded technical robustness, replicate-aware design, and distributional decomposition (G0–G3). G4 blocked mechanistic claims because the adjusted IFN model added little R^2^, entropy-contribution and pseudobulk expression ranks were strongly related, and module conclusions depended on the estimand. G5 was not assessed because no compatible independent paired-donor IFN-β dataset was analyzed. GSE226488 retained only a raw-computation pass at G0; its historical 5000-UMI cap was not real rarefaction, so G1 was placed on hold and downstream gates were not reassessed. These outcomes restrict the conclusion to GSE96583.

## 3. Discussion

This fail-closed reanalysis supports a narrow but reproducible observation within GSE96583: after paired IFN-β stimulation, observed transcript-count diversity increased in CD14+ and FCGR3A+ monocytes and decreased in NK cells, with consistent donor directions and without reversal under real equal-depth sampling, Miller–Madow correction, or either IFN-removal order. The result is an association in a specific public experiment with eight donors. It is not evidence for a universal lineage property, and it does not by itself specify a regulatory mechanism.

The depth audit materially changed the strength and meaning of the evidence. A 5000-UMI cap that affects 157/24,600 cells cannot validate an equal-depth claim. In contrast, multivariate-hypergeometric sampling to 562 UMIs placed every source cell at the same observed molecular depth, and the revised direction was preserved. Agreement with Miller–Madow-corrected estimates provides a separate finite-count sensitivity, although no estimator can recover biological molecules that were not sampled [3]. The 750-UMI results were directionally concordant but were kept secondary because condition-dependent NK retention could itself change the analyzed population. Reporting eligibility is therefore part of the result, not merely a preprocessing detail.

Direct richness, evenness, effective-number, and dominance summaries also changed the interpretation. Detected-gene richness did not show a consistent paired-donor difference in any target lineage. The monocyte entropy increases were instead accompanied by higher normalized evenness and lower top-one and top-three shares. The NK decrease was accompanied by lower evenness and a higher top-three share, while its top-one share was not consistent. These observations describe the sampled transcript composition more precisely than entropy alone, but they should not be translated into claims about cell potency, flexibility, or constrained fate.

The exact IFN/non-IFN identity provides a further guard against a single-score explanation. IFN-associated partition and within-IFN terms were positive in all three lineages. A negative weighted non-IFN term was also present in all three and determined the sign only in NK cells. Thus, the monocyte/NK contrast was a balance of large opposing compositional terms rather than simple presence versus absence of an IFN program. The lineage differences in IFN-set composition—especially the high CXCL10 share in monocytes and broader distribution among IFI6, IFIT genes, MX1, IRF7, and OAS1 in NK cells—offer a descriptive map for follow-up, not proof that any one gene causes the entropy contrast.

The adjusted IFN model corrects an important inferential error in the earlier analysis. Once donor, condition, and technical terms were included, fixed IFN activity added at most 0.0108 R^2^. The previous pooled-cell model mainly compared thousands of non-independent cells across conditions and could not support an independence gate. Likewise, the high rank correlation between entropy contributions and paired pseudobulk log_2_-CPM changes shows that contribution decomposition substantially overlaps conventional expression change. Entropy remains a valid compositional summary, but these data do not show that it reveals a distinct biological process unavailable to expression analysis.

The complete NK contribution distribution argues for similarly restrained wording. Negative contribution mass was diffuse, and the 20 largest negative shifts accounted for only 14.8% of it. Many large negative shifts involved ribosomal transcripts and FTH1, while several canonical IFN-associated and effector transcripts had positive contributions. The observed total decrease therefore cannot be attributed to a small set of cytotoxic genes, nor can it be labeled a functional narrowing. A mechanistic test would require purified and prospectively sampled NK subsets, time-resolved perturbations, functional readouts, and an independent cohort.

The two-part module analysis illustrates why a module entropy value cannot be interpreted without its denominator and zero process. IFN stimulation greatly reduced IFN-module zeros and increased module abundance. Conditional per-cell IFN entropy increased, whereas donor-pseudobulk normalized IFN entropy decreased. Both calculations are correct, but they target different distributions. Moreover, the primary IFN pseudobulk statistic was not exceptional relative to matched random sets in both monocyte lineages. The inflammatory/NF-κB statistic occupied smaller matched-null tails, but this does not show pathway suppression or causal redistribution. These results replace the earlier module-level claim with an estimand-dependent description.

The GSE194122 audit demonstrates the utility of stopping at representation validity. With binary peaks, the implemented ATAC entropy is merely a one-to-one transformation of open-peak count over the observed range. Correlating this value with RNA entropy would mean correlating RNA entropy with a transformed accessibility-depth statistic, not testing diversity of a count-weighted accessibility distribution. Count-weighted fragment information or another observable with independent degrees of freedom would be required. The negative audit does not make a biological statement about RNA–chromatin coordination.

This study has several limitations. The supported contrast comes from one dataset, one stimulation protocol, and eight donors with lupus [13]; no external replication was performed. Generalization to healthy donors or other clinical populations therefore remains untested. The target lineages were fixed from the claims under review rather than used for a discovery-wide screen. Pseudobulk expression differences were descriptive log_2_-CPM contrasts, not a fully specified negative-binomial differential-expression analysis. Gene sets were fixed and audited but remain selective summaries of biology. Rarefaction deliberately discards counts to equalize observed depth, and Miller–Madow correction addresses only one source of entropy-estimation bias. Finally, the current analysis describes observed UMI compositions and cannot separate transcription, RNA stability, capture efficiency, and other processes that contribute to those counts.

An independent dataset capable of assessing G5 should use matched control and IFN-β-stimulated aliquots from at least eight donors not represented in GSE96583, with the three prespecified lineages represented in both conditions for every donor and with raw integer UMI counts plus complete donor, condition, lineage, and library metadata. Eight donors constitute a minimum comparability floor matching the biological-replicate count of GSE96583, not a formal power result; a larger cohort should be prospectively powered for the donor-level contrasts. Because the entropy estimator operates on molecular counts, the auditable depth targets should be expressed as recovered whole-transcriptome UMIs rather than nominal reads per cell: sequencing should permit every QC-passing target cell, under criteria fixed before outcome inspection, to support the locked 562-UMI primary rarefaction. A 750-UMI analysis should remain a sensitivity target and be interpreted only if eligibility is comparable across conditions within every donor-lineage stratum under a tolerance specified in advance. These depths are study-specific replication targets derived from the present audit—562 UMIs retained 24,600/24,600 cells, whereas 750 UMIs exposed condition-imbalanced NK-cell retention—not universal single-cell quality thresholds. Under these conditions, G5 could be evaluated by applying the same donor-level contrasts, estimators, IFN-removal operations, retention reporting, and fail-closed decision rules without outcome-driven modification.

The main methodological lesson is procedural. Validity depended on making the biological replicate explicit, defining equations and operations before rerunning them, verifying identifier semantics, reporting retention, decomposing the statistic, and allowing a failed gate to stop claim advancement. Under these constraints, the dataset supports a bounded lineage-associated contrast, while several stronger and more attractive interpretations fail.

## 4. Materials and Methods

### 4.1. Analysis Governance, Datasets, and Analytical Roles

The revision was executed in a prospective branch separated from the recovered historical analysis. A decision lock specified the unit of inference, contrasts, endpoints, depth controls, gene-set handling, uncertainty summaries, null models, and fail-closed semantics before corrected outputs were inspected. Every generated table was linked to a script and source artifact, and superseded outputs from the identifier-defective run were retained in a clearly marked quarantine directory but excluded from interpretation.

GSE96583 [13] was the sole dataset used for the primary biological contrast. The recovered count object contained 24,600 quality-controlled singlets, 35,635 gene features, matched control and IFN-β-stimulated conditions, eight donors with lupus [13], and published cell-type annotations. The prespecified analysis included CD14+ monocytes, FCGR3A+ monocytes, and NK cells (8949 cells in total). Raw integer UMI counts from the count layer were used; no log-normalized matrix was used to calculate entropy.

GSE194122 [14] was restricted to a representation audit of its recovered binary ATAC object (69,249 cells; 116,490 peaks). It was not used as an external replication of GSE96583. GSE226488 was retained only in the gate ledger as a historical cautionary case; the revision did not reuse its prior depth-controlled biological results because the 5000-UMI operation was invalid and the required donor and cell-type observables were unavailable.

### 4.2. Unit of Inference and Paired Contrasts

For each cell-level metric, cells were first averaged within donor, lineage, and condition. A paired donor contrast was then calculated as

Δ M*_dℓ_* = mean(M)*_dℓ_*_,stim_ − mean(M)*_dℓ_*_,ctrl_,

where *d* indexes donor and *ℓ* indexes lineage. The number of biological replicates was therefore eight. Cells were treated as within-donor subsamples, consistent with replicate-aware single-cell analysis [10,11,12]. A lineage direction was considered technically robust when the mean paired-donor contrast retained its sign across the five prospectively fixed variants and at least six out of eight donors had that sign in every variant. Cell-level values were used for computation and visualization, not to generate biological-replicate *p* values.

### 4.3. Outcome-Blind Symbol–Ensembl Validation

Every symbol–Ensembl pair in the recovered module configurations was compared with the local GSE96583 feature map before the corrected analysis. An identifier was changed only when the requested symbol had one unique exact match in that map. The historical CXCL8/IL8 alias was accepted and flagged. Original identifier, original matrix symbol, corrected identifier, corrected symbol, status, and all symbol candidates were exported. This procedure produced 119 exact configuration rows, 18 unique-match repairs, four accepted alias rows, and zero unresolved rows. The non-IFN exclusion set was frozen as the union of corrected Ensembl identifiers from the pre-existing IFN-response, IFN-response v1, and IFN-response v2 configurations (38 unique genes); no gene was selected from an observed revision effect.

### 4.4. Transcript-Count Diversity, Finite-Count Correction, Richness, and Dominance

For raw UMI count *x*_gc_ for gene *g* in cell *c*, the total count and observed proportion were

N_c_ = ∑_g_ x_gc_,     p_gc_ = (x_gc_)/(N_c_).

The base-2 plug-in Shannon entropy was

H_c_ = −∑_g:xgc>0_p_gc_log_2_ p_gc_.

The observed richness was K_c_ = ∑_g_ 1(x_gc_ > 0). The Miller–Madow correction [3,15] was

H_c_^MM^ = H_c_ + (K_c_ − 1)/(2N_c_ln 2).

We also calculated normalized evenness using the formula

J_c_ = (H_c_)/(log_2_ K_c_)  (K_c_ > 1),

the effective number of expressed genes D_eff,c_ = 2^Hc^, the largest observed transcript share, and the sum of the three largest shares. These companion quantities distinguish changes in detected categories from changes in compositional evenness and dominance.

### 4.5. Hypergeometric Rarefaction and IFN-Removal Order

The historical deterministic 5000-UMI cap was audited but not used as an equal-depth analysis. For corrected rarefaction, molecules were sampled without replacement as follows:

Y_c_^(r)^∼MultiHypergeometric(x_c_,D),     ∑_g_Y_gc_^(r)^ = D.

The primary depth was *D* = 562 UMIs, the minimum whole-transcriptome depth among all 24,600 GSE96583 cells. The sensitivity depth was *D* = 750 among eligible cells. Fifty Monte Carlo draws were generated for each eligible cell and depth. The master seed was 4,476,017; cell-specific seeds were derived from stable cell identifiers so row order could not change the results. Metrics were averaged across the 50 draws within cells before donor aggregation.

IFN dependence was stressed in two orders. For “rarefy then remove,” the complete transcriptome was rarefied to 562 UMIs, the frozen 38-gene IFN union was removed, and residual counts were renormalized. For “remove then rarefy,” the IFN union was removed from raw counts and the residual was rarefied to the prospectively fixed depth of 503 UMIs among eligible cells. Eligibility and retention were reported by donor, lineage, and condition; missing cells were not imputed.

### 4.6. Exact IFN/Non-IFN Chain-Rule Decomposition

Let *I* denote the corrected 38-gene IFN union and

q_c_ = ∑_g∈ I_p_gc_.

For 0 < q_c_ < 1, the proportions within the IFN and complementary partitions were p_gc_/q_c_ and p_gc_/(1 − q_c_), with entropies H_c_(I) and H_c_(Ī). The whole-transcriptome entropy obeys the exact identity

H_c_ = h_2_(q_c_) + q_c_H_c_(I) + (1 − q_c_)H_c_(Ī),

where

h_2_(q) = −qlog_2_q − (1 − q)log_2_(1 − q).

At q = 0 or q = 1, the weighted contribution of the empty partition and the binary partition entropy were defined as zero by continuity, using the convention 0 log_2_0 = 0. Each term was averaged within donor, lineage, and condition before paired contrasts. The reconstructed sum was checked against directly computed H_c_ for every cell.

### 4.7. Transparent IFN Activity, Composition, and Adjusted Models

The fixed primary IFN-v0 set contained 15 genes. For a set *S*, transparent activity was

A_c_(S) = log_2_(1 + 10^6^(∑_g∈ S_x_gc_)/(N_c_)).

This definition has no stochastic or unexported control-gene selection. We reported each gene’s share of donor-pseudobulk IFN-set UMIs by lineage and condition. Leave-one-out analyses removed ISG15, IFIT1, or MX1 individually and jointly.

For raw endpoints, ordinary least-squares base models included donor fixed effects, condition, log(1 + total UMIs), and detected-gene richness. For 562-UMI endpoints, total depth was fixed and the technical term was detected-gene richness. IFN activity was then added to the same base design. We reported base and full R^2^, added R^2^, and the Pearson correlation between outcome and IFN-activity residuals after employing the base model. Donor-level correlations between paired activity and entropy contrasts were descriptive because only eight paired points were available. No threshold was used to promote a biological-independence claim.

### 4.8. Gene-Level Entropy Contributions and Paired Pseudobulk Comparator

The contribution of gene *g* to cell *c* was

C_gc_ = −p_gc_log_2_p_gc_,     H_c_ = ∑_g_ C_gc_.

Contributions were averaged within each donor, lineage, and condition, and paired contrasts were summarized across donors. For the conventional expression comparator, raw counts were summed within donor × lineage × condition. Gene expression was transformed as log_2_(CPM + 1), and stimulated-minus-control differences were paired within donor. Spearman correlation compared the mean gene-level contribution contrast with the mean paired log_2_-CPM contrast. We also calculated overlap and Jaccard similarity between the 50 largest absolute shifts under the two rankings. This was a descriptive comparator, not a negative-binomial differential-expression test.

### 4.9. Two-Part Module Analysis and Matched Nulls

The primary modules were the pre-existing 15-gene IFN-response set and nine-gene inflammatory/NF-κB set. Pre-existing expanded IFN-response v1 (31 genes), IFN-response v2 (18), TNF/NF-κB v1 (26), and inflammatory-response v1 (17) sets were sensitivities [16]. For module *S*, module UMIs, module detection, and the fraction of module-zero cells were reported before entropy. Among cells with positive module counts,

H_c_(S) = −∑_g∈ S_(x_gc_)/(∑_j∈ S_x_jc_) log_2_((x_gc_)/(∑_j∈ S_x_jc_)).

We reported conditional H_c_(S), H_c_(S)/log_2_|S|, and detected-gene evenness where defined. For the donor-pseudobulk estimand, module counts were summed across cells within donor, lineage, and condition before entropy calculation; the primary normalized statistic was pseudobulk H(S)/log_2_|S|.

For each primary module and monocyte lineage, 500 random gene sets of the same size were assembled. Candidate genes had at least one observed UMI in that lineage and did not belong to the target module. Each target gene was matched using deciles of control-cell mean expression and detection prevalence; matching radius was expanded only when required to obtain an adequate pool. Sampling was without replacement where possible and used seed 4,476,017. The null statistic was the median paired-donor contrast in pseudobulk normalized module entropy. The empirical percentile and two-sided tail area were descriptive.

### 4.10. ATAC Structural Audit

The recovered GSE194122 ATAC calculation encoded each peak as closed or open. If *m*_c_ of *P* peaks were open, the implemented value was

H_ATAC,c_ = h_2_((m_c_)/(P)), P = 116,490.

We checked, across all 69,249 cells, equality between stored total ATAC counts and open-peak number, Spearman correlation between H_ATAC_ and open-peak number, and absolute error from the formula. Matrix nonzero values were inspected in 512 sampled rows without materializing the complete sparse matrix. A deterministic relationship was specified in advance as a representation failure for cross-modal entropy coupling.

### 4.11. Uncertainty Summaries and Fail-Closed Gates

For each donor-paired endpoint we reported *n*, the mean, the median, positive and negative direction counts, an exact two-sided sign-flip probability for the mean (all 2^8^ sign assignments when eight donors were available), and a 95% percentile interval from 10,000 deterministic donor bootstrap resamples. Given eight donors, intervals and direction counts were given priority over binary declarations of statistical significance. No missing donor–condition pair was imputed.

The sequential gates were: G0, data and representation validity; G1, technical robustness; G2, replicate-aware design; G3, distributional decomposition; G4, alternative-explanation stress test; and G5, external replication. Following a required failure, downstream advancement gates were recorded as not assessed. A diagnostic calculation after failure could be reported only as contextual information.

### 4.12. Software and Reproducibility

The corrected analyses used Python 3.10.20, NumPy 2.2.6, pandas 2.3.3, SciPy 1.15.3, anndata 0.11.4, and Matplotlib 3.10.8. All stochastic operations used the frozen master seed 4,476,017 and stable, labeled sub-seeds. Machine-readable outputs, run records, source hashes, the corrected gene-set configuration, and the gate ledger were included in the revision bundle.

## 5. Conclusions

In GSE96583, paired IFN-β stimulation was associated with robust increases in observed transcript-count Shannon diversity in two monocyte lineages and a decrease in NK cells. The differences were accompanied by changes in evenness and dominance, whereas richness changes were not consistent across donors. Exact decomposition revealed positive IFN-associated terms opposed by a negative non-IFN term in every lineage. Donor-aware modeling, overlap with pseudobulk expression changes, module-estimand dependence, and the binary-ATAC representation audit set firm limits on interpretation. The result should be described as a donor-consistent, single-dataset association; claims of mechanism, entropy information independent of expression change, biological RNA–ATAC coupling, or cross-dataset generality are not supported.

## Figures and Tables

**Table 1:** Donor-paired whole-transcriptome entropy robustness. Values are mean stimulated-minus-control changes in bits. Brackets are 95% percentile intervals from 10,000 donor bootstrap resamples for raw plug-in entropy. MM, Miller–Madow; IFN, corrected 38-gene union.

Lineage	Raw Plug-in ΔH [95% Interval]	Raw MM	Rarefied 562	Rarefied 562 + MM	Rarefy 562 → Remove IFN	Remove IFN → Rarefy 503	Donor Direction Across Five Primary Variants
CD14+ monocytes	+0.138 [+0.075, +0.201]	+0.111	+0.145	+0.129	+0.113	+0.194	≥7/8 positive
FCGR3A+ monocytes	+0.329 [+0.287, +0.372]	+0.315	+0.330	+0.333	+0.297	+0.364	8/8 positive
NK cells	−0.080 [−0.103, −0.057]	−0.100	−0.117	−0.130	−0.176	−0.143	8/8 negative

**Table 2:** Exact IFN/non-IFN chain-rule decomposition. Values are mean paired-donor stimulated-minus-control changes in bits, except Δ IFN fraction.

Lineage	Δ IFN Fraction	Δ Binary Partition Term	Δ Weighted Within-IFN Term	Δ Weighted Non-IFN Term	Reconstructed ΔH
CD14+ monocytes	+0.1229	+0.502	+0.387	−0.751	+0.138
FCGR3A+ monocytes	+0.1005	+0.421	+0.338	−0.430	+0.329
NK cells	+0.0399	+0.215	+0.145	−0.440	−0.080

## Data Availability

The source datasets are publicly available under GEO accessions GSE96583, GSE194122, and GSE226488. GSE96583 was the primary revised analysis; GSE194122 was used only for the ATAC representation audit; GSE226488 was retained as a held historical case and was not biologically reassessed in this revision. No new primary data were generated. Corrected analysis scripts, configuration files, gene-set manifests, retained run records, derived machine-readable outputs and SHA-256 checksums are provided in Supplementary Code S1. Supplementary Tables S1–S6 and Figures S1–S3 provide the supporting results, with a worksheet index included. Large RNA and ATAC matrices are not redistributed in Code S1; its input manifest documents the source accessions, required analysis objects and recorded checksums. The historical v0.3 Zenodo record (version DOI: 10.5281/zenodo.20142865; concept DOI: 10.5281/zenodo.20142864) is not the archive of this corrected code.

## Supplementary Materials

The following supporting information can be downloaded at: https://www.mdpi.com/article/10.3390/ijms27188182/s1. The archive includes Tables S1–S6 (with a worksheet index), Figures S1–S3 and Supplementary Code S1 (corrected analysis code and reproducibility records).
